# *Lycium barbarum* Polysaccharides Improve Testicular Spermatogenic Function in Streptozotocin-Induced Diabetic Rats

**DOI:** 10.3389/fendo.2020.00164

**Published:** 2020-04-17

**Authors:** Xiaocan Lei, Peng Huo, Yaohui Wang, Yuanjie Xie, Qingxiang Shi, Haoyan Tu, Jun Yao, Zhongcheng Mo, Shun Zhang

**Affiliations:** ^1^Department of Histology and Embryology, Clinical Anatomy & Reproductive Medicine Application Institute, University of South China, Hengyang, China; ^2^School of Public and Health, Guilin Medical University, Guilin, China; ^3^School of Basic Medicine, Zunyi Medical University, Zunyi, China; ^4^Department of Reproductive Medical Center, The Affiliated Hospital of Guilin Medical University, Guilin, China

**Keywords:** LBP, diabetes, testis, PCNA, SIRT1, HIF-1α

## Abstract

The objective of this study was to investigate the protective effects of *Lycium barbarum* polysaccharides (LBP) on testicular spermatogenic function in streptozotocin (STZ)-induced diabetic rats. Compared to the control group, blood glucose levels were significantly increased and the insulin resistance was markedly aggravated in STZ-induced diabetic rats. Further, the weight of testis and epididymis and the sperm number and motility were decreased in diabetic rats. Pathological changes were also observed in the spermatogenic tubules, along with a decreased number of spermatogenic cells, downregulated proliferating cell nuclear antigen (PCNA) expression, and increased cell apoptosis in the testes. Compared to the saline-treated diabetic rat group, metformin and LBP treatment significantly decreased the level of blood glucose and improved insulin resistance and testicular function. After treatment with metformin and LBP, the pathological changes in the spermatogenic tubules improved significantly, with an increase in the number of spermatogenic cells, upregulation of PCNA, and suppression of apoptosis in the testes. The expressions of sirtuin 1 (SIRT1) and hypoxia-inducible factor 1-alpha (HIF-1α) in diabetic testes were also upregulated by metformin or LBP treatment. In summary, LBP exerted protective effects by increasing cell proliferation, inhibiting cell apoptosis, and regulating SIRT1/HIF-1α expression in the testes of diabetic rats.

## Introduction

Diabetes mellitus (DM) is a metabolic disorder characterized by chronic hyperglycemia due to defects in insulin action and secretion (1, 2). In China, the prevalence of DM is about 9.7% in the population >18 years of age and about 20% in the population over 60 years of age (3). DM-associated complications include nephropathy, neuropathy, retinopathy, cardiovascular diseases, and metabolic syndromes (4). In the male reproductive system, glucose homeostasis is important for spermatogenesis. There is growing evidence showing that DM can cause damage to the male reproductive system, including decreased testicular weight and sperm number and motility and increased abnormal sperm number (5). So far, the molecular mechanisms underlying DM-induced male reproductive dysfunction remain elusive.

*Lycium barbarum* has long been well-known as a traditional Chinese medicine that promotes health and longevity and is mainly cultivated in the Ningxia district of China (6, 7). Studies have shown that *Lycium barbarum* polysaccharides (LBP) possess multiple pharmacological functions including immunomodulatory, antioxidant, hypolipidemic, anti-tumor, and anti-aging functions (8–12). Recently, several lines of evidence have indicated the protective effects of LBP on the male reproductive damage induced by radiation (13), chemotherapeutic reagents (14, 15), nonylphenol (16), and corticosterone (17). In a diabetic animal model, LBP could attenuate diabetic testicular dysfunction *via* inhibition of the PI3K/Akt pathway-mediated abnormal autophagy in male mice (18). Shi et al. showed that LBP could exert protective effects on DM-induced spermatogenic dysfunction by increasing antioxidative enzyme activities and inhibiting cell death (19). In addition, LBP could exert functional recovery of male sexual dysfunction and fertility damage induced by DM in male mice by regulating the hypothalamus-pituitary-gonadal axis endocrine activity (20). However, detailed investigation into the molecular mechanisms underlying LBP-mediated protective effects on male reproductive dysfunction induced by DM is required.

Sirtuin 1 (SIRT1) belongs to the mammalian superfamily of sirtuins (21). SIRT1 has been identified to be upregulated under diabetic conditions, and it can regulate glucose metabolism *via* deacetylase activity on respective targets (21). In pancreatic β-cells, SIRT1 can promote the secretion of insulin and protect cells against oxidative stress and inflammation (21). Additionally, studies have found that SIRT1 was significantly decreased in infertile oligoasthenoteratozoospermic men with varicocele (22, 23). In animal studies, SIRT1 was reported to regulate acrosome biogenesis by modulating autophagic flux during spermiogenesis in mice (24). Recent findings revealed that ferulic acid protects male rats against radiation-induced testicular damage by increasing SIRT1 expression (25). Existing evidence indicates the importance of SIRT1 in the male reproductive system. Hypoxia-inducible factor 1-alpha (HIF-1α) is a hypoxia-activated transcription factor that confers protective effects in hypoxic conditions (26). Studies have shown the dysregulated expression of HIF-1α under diabetic conditions (27). Testis is a relatively hypoxic tissue, and HIF-1α regulates the primary transcriptional responses to hypoxic stress in normal and transformed cells, which play an adaptive role in conferring protection against cell death in the testes (28). Studies have also reported that SIRT1 deacetylates and stabilizes HIF-1α through direct interactions (29), implying that the SIRT1/HIF-1α axis may be an important mediator in protection against male reproductive dysfunction induced by DM.

In the present study, we aimed to determine the protective effects of LBP on spermatogenic dysfunction in streptozotocin (STZ)-induced diabetic rats using immunohistochemical analysis of proliferating cell nuclear antigen (PCNA) expression, cell apoptosis, sirtuin 1 (SIRT1), and hypoxia-inducible factor 1-alpha (HIF-1α) expression in the testes. The present study may further advance our understanding of molecular mechanisms of LBP-mediated protective effects on DM-induced male reproductive dysfunction.

## Materials and Methods

### Preparation of LBP and Metformin

LBP was prepared based on previous methods (9) and was purchased from Xi‘an Xiaocaokeji Ltd. (Xi', China). Metformin was purchased from Sigma-Aldrich (St. Louis, USA). LBP and metformin were dissolved in saline for oral administration.

### Animals and Induction of Diabetes

Male Sprague-Dawley rats (4–6 weeks old, body weight 170 ± 10 g) were purchased from the Animal Experimental Center of Daping Hospital, Third Military Medical University. All experimental procedures were approved by the Animal Ethics Committee of Zunyi Medical University. For the induction of diabetes, rats were fed a high-fat diet for 6 weeks and then intraperitoneally injected with 45 mg/kg STZ (dissolved in 0.1 M citrate buffer at pH 4.5). The fasting blood glucose levels were monitored for 3 consecutive days after STZ injection, and rats with fasting glucose levels ≥ 16.7 mM were considered diabetic.

### Treatment Groups

Rats were randomly divided into four groups: the control group (control group, *n* = 6), DM + saline group (model group, *n* = 6), DM + metformin group (*n* = 6), and DM + LBP group (*n* = 6). For the control group, rats were fed a basal diet for 6 weeks and were then orally administered saline (1 mL/kg/d). For the DM + saline group, diabetic rats were orally administered saline (1 mL/kg/d). For the DM + metformin group, diabetic rats were orally administered metformin (100 mg/kg/d). For the DM + LBP group, diabetic rats were orally administered LBP (100 mg/kg/d). All the rats were fed with a basal diet during the drug treatments, and after a 6 week treatment, rats were sacrificed, and relevant tissues were collected for further analysis.

### Measurement of Blood Glucose and Insulin Levels and Homeostatic Model Assessment of Insulin Resistance (HOMA-IR)

One day before the end of the experiments, animals were fasted overnight, and then blood glucose levels were detected using a Glucose Meter (Contournext, Parsippany, USA), while blood insulin levels were detected using a commercial insulin kit (Abcam, Cambridge, USA). HOMA-IR was calculated based on the following formula: (fasting blood glucose levels × fasting blood insulin levels)/22.5.

### Assessment of Sperm Number and Motility

Sperm samples were collected from the testicular tissues and incubated in saline at 37°C. Sperm number and motility were evaluated using a computer-aided sperm analysis system (Sperm Class Analyzer, MICROPTIC, Barcelona, Spain).

### Histopathological Examination

The testicular tissues were dissected and fixed in 4% paraformaldehyde overnight at 4°C, embedded in paraffin, sectioned into 5-μm-thick slices, deparaffinized, and stained with hematoxylin and eosin. The morphology of the testes and the number of spermatogonia and spermatocytes were evaluated under a light microscope.

### Immunohistochemical Analysis of Proliferating Cell Nuclear Antigen (PCNA), Sirtuin 1 (SIRT1), and Hypoxia-Inducible Factor 1-Alpha (HIF-1α) Expression

The 5-μm-thick deparaffinized slices were rehydrated using 100 to 75% ethanol. Antigen retrieval was performed by incubating the slices with sodium citrate buffer with an application of high voltage for 3 min. Then, the slices were incubated with 3% H_2_O_2_-methanol solution at room temperature for 10 min. The slices were blocked with 5% bovine serum albumin in 1% Triton X-100 phosphate buffered saline (PBS) at room temperature for 1 h, followed by incubation with PCNA, SIRT1, and HIF-1α primary antibodies at 4°C overnight. After washing the sections with PBS thrice for 5 min each, the slices were incubated with biotinylated anti-rabbit immunoglobulin G at room temperature for 1 h. The slices were then incubated with avidin-biotin-peroxidase complexes. The antigens were visualized after incubation with DAB solution. The number of PCNA-, SIRT1- and HIF-1α-positive cells were evaluated under a light microscope.

### Terminal Deoxynucleotidyl Transferase-Mediated dUTP Nick-End Labeling (TUNEL) Assay

Apoptosis in testicular tissues was evaluated by TUNEL assay. The 5-μm-thick deparaffinized slices were rehydrated using 100 to 75% ethanol. The slices were incubated with 1% Triton X-100 at room temperature for 30 min and were then incubated with 3% H_2_O_2_-methanol solution at room temperature for 10 min. After that, proteinase K was added to the slices, and they were incubated for 15 min. The slices were incubated with TdT-enzyme at 37°C for 1 h and washed with PBS thrice for 5 min each. Then, 100 μL of digoxigenin (conjugated to horseradish peroxidase, POD) was placed on each section; 3, 3′-Diaminobenzidine (DAB) was used as a staining agent. Apoptotic cells were stained brown and analyzed by randomly counting TUNEL-positive cells.

### Quantitative Real-Time Polymerase Chain Reaction (qRT-PCR)

Total RNA from testicular tissues was extracted using TRIzol reagent (Invitrogen, Carlsbad, USA). RNA was reverse transcribed into cDNA using the PrimeScript 1st strand cDNA Synthesis Kit (Takara Bio, Dalian, China). Real-time PCR was performed on an ABI7900 PCR system (Applied Biosystems, Foster City, USA) using SYBR® Green Real-Time PCR Master Mix (Takara Bio). GAPDH was used as an internal control for relevant mRNA expression. The relative expression of PCNA mRNA was calculated using the comparative Ct method. The primer sequences are shown in Table 1.

**Table 1:** Primer sequences of target genes.

**Gene**	**Sequences (5^**′**^-3^**′**^)**
PCNA	F: GCTCCATCCTGAAGAAGGT
	R: TGCACTAAGGAGACGTGAGA
Bax	F: GAGACACCTGAGCTGACCTT
	R: TCCATGTTGTTGTCCAGTTC
Bcl-2	F: AGTACCTGAACCGGCATCT
	R: TCTTCAGAGACAGCCAGGA
Caspase-3	F: CCGGTTACTATTCCTGGAGA
	R: TAACACGAGTGAGGATGTGC
SIRT1	F: GTGGCAGTAACAGTGACAGTG
	R: GTCAGCTCCAGATCCTCCAG
GAPDH	F: CCTCAAGATTGTCAGCAATG
	R: CAGTCTTCTGAGTGGCAGTG

### Statistical Analysis

All data analyses were performed using GraphPad Prism (Version 6.0, GraphPad Software, La Jolla, USA). Data are presented as mean ± standard deviation. Significant differences among different treatment groups were analyzed using one-way ANOVA followed by Dunnett's *post-hoc* test. *P* < 0.05 was considered statistically significant.

## Results

### Effects of Metformin and LBP Treatments on Body Weight, Blood Glucose, Insulin Levels, and Insulin-Resistant Index in Diabetic Rats

Initially, we observed that saline-treated diabetic rats had lower body weight than the controls and that metformin treatment increased body weight in diabetic rats when compared to saline-treated diabetic rats. However, LBP treatment failed to significantly increase body weight compared to saline-treated diabetic rats (Table 2). Before any treatment, diabetic rats had higher fasting glucose levels than the control group, but metformin and LBP treatments both reduced fasting glucose levels compared to saline-treated diabetic rats (Table 2). Saline-treated diabetic rats had higher fasting insulin levels than the controls, while metformin and LBP treatments further increased fasting insulin levels compared to saline-treated diabetic rats (Table 2). In addition, the increased insulin resistance index in diabetic rats was alleviated by treatment with metformin or LBP (Table 2).

**Table 2:** Effects of metformin and LBP treatments on the body weight, blood glucose, insulin levels, and insulin-resistant index in diabetic rats.

**Group**	**Body weight (g)**		**Fasting blood glucose (mM)**		**Fasting insulin (μIU/mL)**	**Insulin resistance index**
	**Pre-induction**	**After treatment**	**Pre-treatment**	**After treatment**		
Control	329.3 ± 6.732	389.7 ± 9.234	6.358 ± 0.4870	5.112 ± 0.2141	71.35 ± 12.48	21.87 ± 2.33
DM + saline	321.8 ± 15.14	234.9 ± 21.18^*^	21.48 ± 0.6938^*^	28.53 ± 0.7424^*^	99.88 ± 22.24^*^	178.8 ± 48.62^*^
DM + Metformin	328.9 ± 8.412	348.7 ± 11.25^*^^#^	19.255 ± 0.4487^*^	5.354 ± 0.2388^#^	177.45 ± 41.28^*^^#^	41.56 ± 14.14^#^
DM + LBP	322.0 ± 7.225	294.2 ± 17.34^*^^$^	23.77 ± 1.4520^*^	7.600 ± 1.6000^#^	143.20 ± 26.28^*^^#^	50.23 ± 19.06^#^

* P < 0.05 vs. Control;

# P < 0.05 vs. DM + saline group;

$ *P < 0.05 vs. DM + Metformin group*.

### Effects of Metformin and LBP on Testis and Epididymis Weight, Sperm Number, and Motility

As shown in Table 3, the saline-treated diabetic rats had lower testis and epididymis weight and also reduced sperm number and motility compared to controls (Table 3). Both metformin and LBP treatments significantly increased the testis and epididymis weight and also increased sperm number and motility compared to saline-treated diabetic rats (Table 3).

**Table 3:** Effects of metformin and LBP on testis and epididymis weight and sperm number and motility.

**Group**	**Testis weight (g)**	**Epididymis weight (g)**	**Sperm number (× 10^**6**^)**	**Sperm motility (%)**
Control	1.698 ± 0.058	0.7144 ± 0.0980	149.8 ± 11.42	11.60 ± 5.400
DM + saline	1.211 ± 0.121^*^	0.3599 ± 0.0521^*^	55.22 ± 10.99^*^	3.300 ± 1.500^*^
DM + Metformin	1.662 ± 0.049^#^	0.5774 ± 0.0994^#^	139.4 ± 66.20^#^	14.25 ± 13.15^#^
DM + LBP	1.664 ± 0.062^#^	0.5334 ± 0.0396^#^	137.0 ± 31.30^#^	12.17 ± 3.440^#^

* P < 0.05 vs. Control;

# P < 0.05 vs. DM + saline group.

### Effects of Metformin and LBP Treatment on the Number of Spermatogonia and Spermatocytes in Diabetic Rats

**Figure 1:**
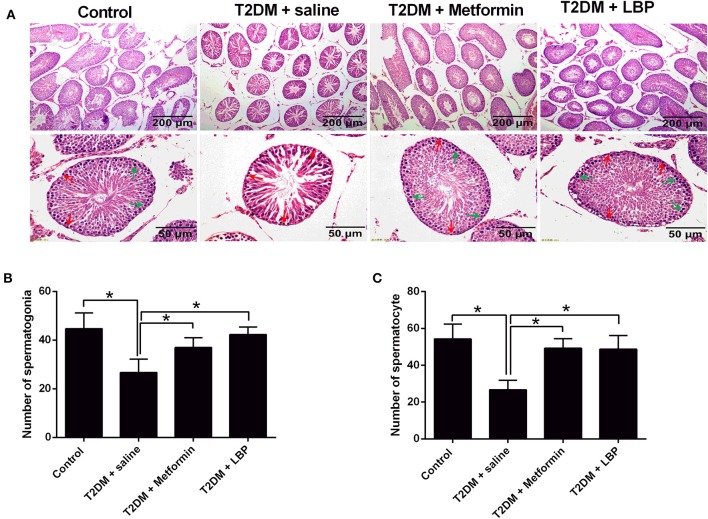
Effects of metformin and LBP treatment on the number of spermatogonia and spermatocytes in diabetic rats. **(A)** Hematoxylin and eosin staining of testicular tissue from control rats and diabetic rats treated with saline, metformin, or LBP. **(B)** Quantification of spermatogonia in testicular tissues from different treatment groups. **(C)** Quantification of spermatocytes in testicular tissues from different treatment groups. *N* = 6. Significant differences between treatment groups are indicated as *P < 0.05.

In the saline-treated diabetic rats, the seminiferous tubules were atrophic, with only 1–3 layers of cells in a disordered arrangement with many vacuoles; the spermatogenic cells were sparsely arranged, and the sperm number was largely reduced (Figure 1A). Metformin and LBP treatments both attenuated the pathological changes in the seminiferous tubules of diabetic rats (Figure 1A). Quantification of the spermatogonia and spermatocytes showed that the saline-treated diabetic rats had a reduced number of spermatogonia and spermatocytes compared to the controls (Figures 1B,C), while metformin and LBP treatments both increased the number of spermatogonia and spermatocytes in the testicular tissues compared to saline-treated diabetic rats (Figures 1B,C).

### Effects of Metformin and LBP Treatment on PCNA Expression in Testis From Diabetic Rats

**Figure 2:**
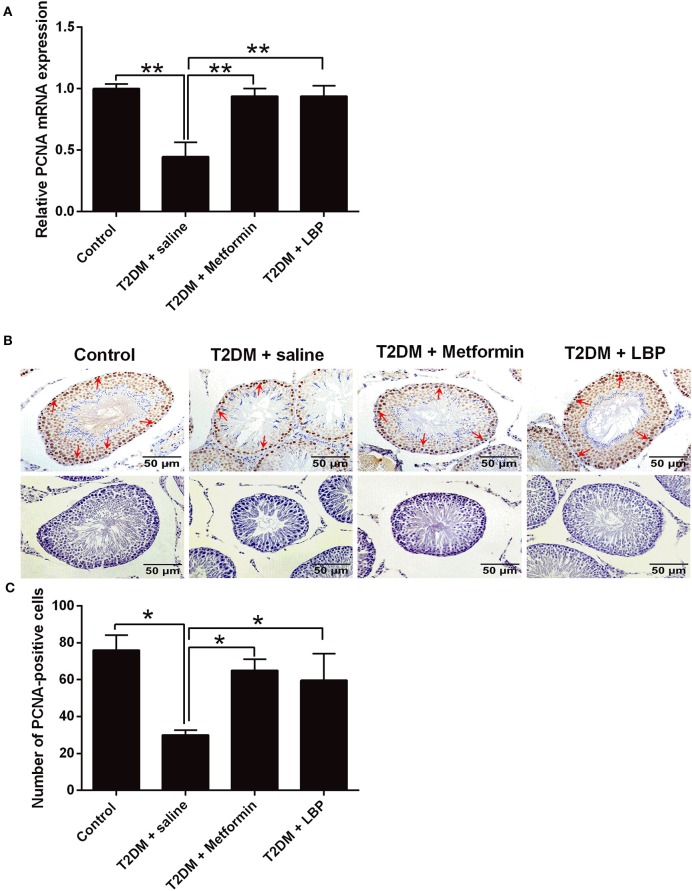
Effects of metformin and LBP treatment on PCNA expression in testicular tissues of diabetic rats. **(A)** qRT-PCR analysis of PCNA mRNA expression in testis from control rats and diabetic rats treated with saline, metformin, or LBP. **(B)** Immunohistochemistry analysis of PCNA expression in testis from control rats and diabetic rats treated with saline, metformin, or LBP. Slices in the upper row show PCNA-positive cells; slices in the lower row are negative controls. **(C)** Quantification of PCNA-positive cells in testis from different treatment groups. *N* = 6. Significant differences between treatment groups are indicated as *P < 0.05 and **P < 0.01.

PCNA mRNA expression levels in the testicular tissues from different treatment groups were evaluated by qRT-PCR. We observed that the mRNA expression was significantly suppressed in the saline-treated diabetic rats compared to the control group and that metformin and LBP treatments both increased PCNA mRNA expression in diabetic rats (Figure 2A). Immunohistochemically determined PCNA protein expression in the testicular tissues from different treatment groups and from diabetic rats treated with saline showed a decrease in the number of PCNA-positive cells in the testicular tissues compared to the control group (Figures 2B,C). Consistently, metformin and LBP treatments both increased the number of PCNA-positive cells in the testicular tissues of diabetic rats compared with saline treatment (Figures 2B,C).

### Effects of Metformin and LBP Treatment on Apoptosis in Testicular Tissues From Diabetic Rats

**Figure 3:**
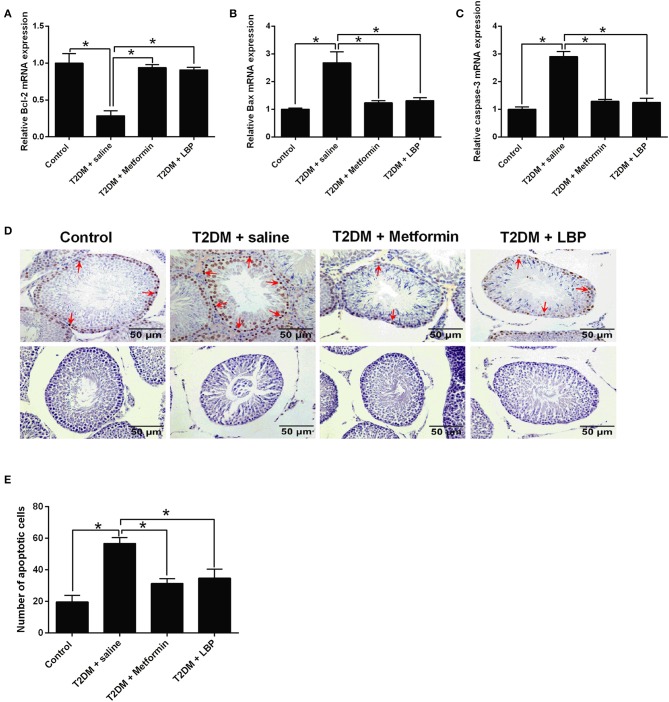
Effects of metformin and LBP treatment on the cell apoptosis in testis from diabetic rats. **(A–C)** qRT-PCR analysis of Bcl-2, Bax, and caspase-3 expression in testis from control rats and diabetic rats treated with saline, metformin, or LBP. **(D)** TUNEL assay analyzed the apoptotic cells in testis from control rats and diabetic rats treated with saline, metformin, or LBP. Slices in the upper row show apoptotic cells; slices in the lower row are negative controls **(E)** Quantification of apoptotic cells in testis from different treatment groups. *N* = 6. Significant differences between treatment groups are indicated as *P < 0.05.

The qRT-PCR results showed that Bcl-2 mRNA was downregulated and the mRNA expressions of Bax and caspase-3 were upregulated in the saline-treated diabetic rats when compared to the control group. Metformin and LBP treatments attenuated the DM-induced changes in the apoptosis-related mediators (Figures 3A–C). Using TUNEL assay, we identified an increase in the number of apoptotic cells in the testicular tissues from different treatment groups and from diabetic rats with saline treatment compared to the control group (Figures 3D,E). Consistently, metformin and LBP treatments both decreased the number of apoptotic cells in the testicular tissues of diabetic rats compared with saline treatment (Figures 3D,E).

### Effects of Metformin and LBP Treatment on SIRT1 Expression in Testicular Tissues From Diabetic Rats

**Figure 4:**
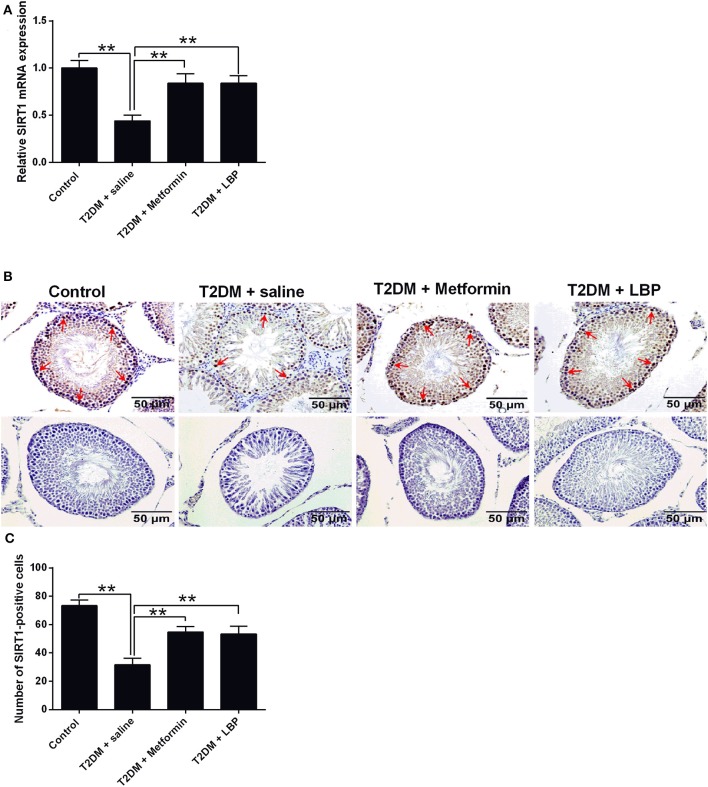
Effects of metformin and LBP treatment on SIRT1 expression in testis from diabetic rats. **(A)** qRT-PCR analysis of PCNA mRNA expression in the testis from control rats and diabetic rats treated with saline, metformin, or LBP. **(B)** Immunohistochemistry analysis of SIRT1 expression in the testis from control rats, and diabetic rats treated with saline, metformin, or LBP. Slices in the upper row show SIRT1-positive cells; slices in the lower row are negative controls **(C)** Quantification of SIRT1-positive cells in testis from different treatment groups. *N* = 6. Significant differences between treatment groups are indicated as **P < 0.01.

SIRT1 mRNA expression was significantly suppressed in the saline-treated diabetic rats compared to the control group, and metformin and LBP treatments both increased SIRT1 mRNA expression in diabetic rats (Figure 4A). Immunohistochemical analysis of SIRT1 in the testes from different treatment groups and from diabetic rats treated with saline showed a decrease in the number of SIRT1-positive cells in the testicular tissues compared to the control group (Figures 4B,C). Consistently, metformin and LBP treatments both increased the number of SIRT1-positive cells in the testicular tissues of diabetic rats when compared to treatment with saline (Figures 4B,C).

### Effects of Metformin and LBP Treatment on HIF-1α Expression in Testicular Tissues From Diabetic Rats

**Figure 5:**
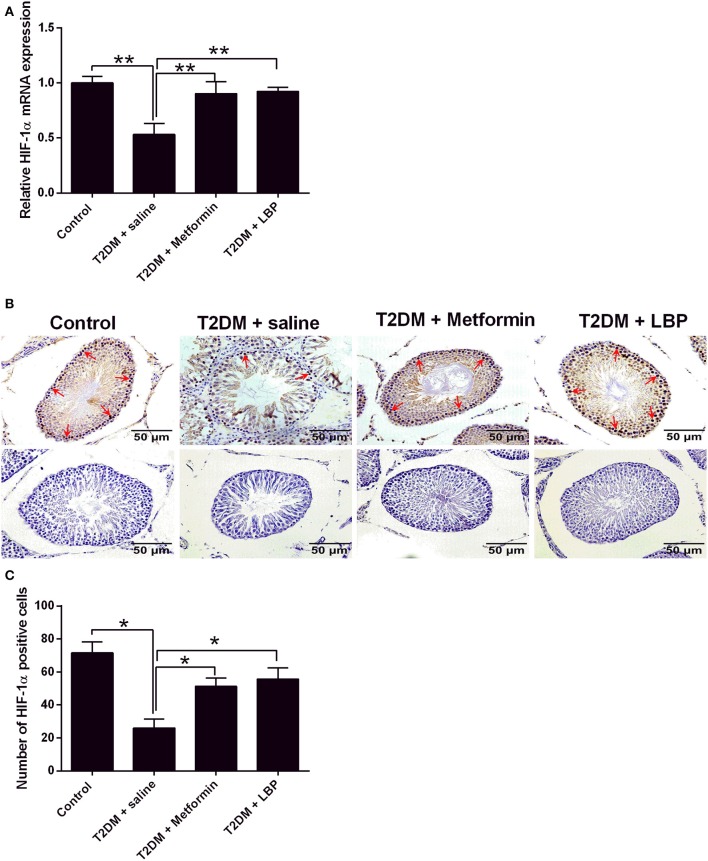
Effects of metformin and LBP treatment on HIF-1α expression in testis from diabetic rats. **(A)** qRT-PCR analysis of HIF-1α mRNA expression in testis from control rats and diabetic rats treated with saline, metformin, or LBP. **(B)** Immunohistochemistry analysis of HIF-1α expression in testis from control rats and diabetic rats treated with saline, metformin, or LBP. Slices in the upper row show HIF-1 α -positive cells; slices in the lower row are negative controls. **(C)** Quantification of HIF-1α-positive cells in testis from different treatment groups. *N* = 6. Significant differences between treatment groups are indicated as *P < 0.05 and **P < 0.01.

The mRNA expression of HIF-1α was downregulated in the testicular tissues of saline-treated diabetic rats compared to the control group (Figure 5A). Metformin and LBP treatments both significantly upregulated HIF-1α mRNA expression in the testicular tissues compared to saline-treated diabetic rats (Figure 5A). Immunohistochemical analysis of HIF-1α protein expression in the testicular tissues from the different treatment groups and diabetic rats with saline showed a decrease in the number of HIF-1α-positive cells in the testicular tissues compared to the control group (Figures 5B,C). Consistently, metformin and LBP treatments both increased the number of HIF-1α-positive cells in the testicular tissues of diabetic rats compared with the control group (Figures 5B,C).

## Discussion

LBP is a water-soluble polysaccharide extracted from *Lycium barbarum* and exerts multiple biological functions including protective effects on the male reproductive system (30). In this study, our results showed that STZ-induced diabetic rats showed impaired spermatogenic function with a decrease in PCNA, SIRT1, and HIF-1α expression and an increase in cell apoptosis in the testes. Both metformin and LBP treatments increased the expression of PCNA and inhibited cell apoptosis in the testes of diabetic rats. In addition, the expressions of SIRT1 and HIF-1α in diabetic testes were also upregulated by metformin and LBP treatments.

The molecular mechanisms of LBP-mediated protective effects on the male reproductive system have been elucidated in several studies. It has been found that LBPs could reduce irradiation-induced spermatogenic cell apoptosis by upregulating Bcl-2 expression and decreasing Bax expression and mitochondrial potential (31). In addition, LBP could recover serum testosterone levels, increase superoxide dismutase activity, decrease malondialdehyde levels, promote oxidative balance, and rescue testicular DNA damage in irradiated rats (13). LBP could also improve sperm density and motility, increase the rate of normal sperm morphology, enhance superoxide dismutase activity, and decrease the nitrate nitrogen level in testes of mice treated with cyclophosphamide (15). In STZ-induced diabetic male mice, LBP could enhance spermatogenesis by increasing antioxidant activity and inhibiting apoptosis in the testes (19). Consistently, our data showed the LBP treatment could significantly decrease the level of blood glucose, improve insulin resistance, and increase the weight of testis and epididymis as well as the number and motility of sperm. After LBP treatment, the pathologic changes in the spermatogenic tubules improved significantly, with increased numbers of spermatogenic cells. LBP treatment significantly upregulated PCNA expression at both the mRNA and protein levels in the testes of diabetic rats. Moreover, LBP treatment also reduced cell apoptosis in the testes of diabetic rats by downregulating Bax and caspase-3 expression and upregulating Bcl-2 expression. Collectively, our data indicate that LBP exerts protective effects against DM-induced male reproductive damage by increasing cell proliferation and inhibiting cell apoptosis in the testis.

SIRT1 belongs to the mammalian Sirtuin gene family and functions as an NAD-dependent histone deacetylase. Notably, SIRT1 plays a key role in regulating glucose metabolism (32). DM-induced germ cell apoptosis was associated with a decrease in SIRT1 expression in male mice (33), and *Sirt1* knockdown impaired spermatogenesis and germ cell function (34). Our results showed that DM induced the downregulation of SIRT1 in the testes of diabetic rats and that LBP treatment increased the mRNA and protein expression levels of SIRT1 in the testes of diabetic rats. Our findings are in agreement with previous studies showing that LBP enhanced SIRT1 expression in hypoxic pulmonary vascular smooth muscle cells and in high-fat-diet-induced hepatic steatosis (35, 36). The testis is a relatively hypoxic tissue, and HIF-1α regulates the primary transcriptional responses to hypoxic stress in normal and transformed cells, which plays an adaptive role in conferring protection against cell death in the testes (28). In addition, SIRT1 stabilizes HIF-1α by direct binding and deacetylation during hypoxia (29). In the present study, we showed that DM-induced downregulation of HIF-1α expression in the testes, and LBP treatment increased HIF-1α expression levels in the testes of diabetic rats. Collectively, these results imply that LBP exerted protective effects on DM-induced reproductive injury by regulating SIRT1 and HIF-1α expression in the testes. Our study proposes that LBP could increase the expression of SIRT1, which results in the stabilization of HIF-1α in the testis under diabetic conditions, and that the upregulated HIF-1α expression may confer testicular production in diabetic rats.

Metformin has been widely used in the treatment of type 2 diabetes; however, metformin treatment is often associated with side effects including nausea, vomiting, diarrhea, and other gastrointestinal reactions, which limits its use for treating diabetic complications (37, 38). Our results showed that the protective effect on diabetic testicular injury is comparable to that of metformin. Because of the rarity of side effects of LBP, it may represent a better strategy for managing male reproductive dysfunction in DM. In this study, we used STZ in combination with a high-fat diet to induce diabetes in the animals; thus, the model used in this study can be considered as a mixed model of type 1/type 2 diabetes. In future studies, we would use proper mouse strains to induce type 2 diabetes and to further examine the protective effects of LBP on the male reproductive system under type 2 diabetic conditions. The findings of this study are still in the preliminary stages, and the underlying molecular mechanisms are still unclear and require further investigation.

In conclusion, our results indicated the protective effects of LBP on the DM-induced male reproductive damage in rats, and mechanistic studies showed that LBP exerted these protective effects by increasing cell proliferation, inhibiting cell apoptosis, and regulating SIRT1/HIF-1α expression in the testes of diabetic rats.

## Data Availability Statement

The raw data supporting the conclusions of this article will be made available by the authors, without undue reservation, to any qualified researcher.

## Ethics Statement

All of the experimental procedures were approved by the Animal Ethics Committee of Zunyi Medical University.

## Author Contributions

XL, SZ, and ZM designed the whole study. XL, PH, YX, HT, and JY performed the experiments and analyzed the data. YW and QS performed the statistical analysis. SZ and ZM wrote the manuscript. All authors approved the manuscript for submission.

## Conflict of Interest

The authors declare that the research was conducted in the absence of any commercial or financial relationships that could be construed as a potential conflict of interest.

## References

[1] Atkinson MA Eisenbarth GS Michels AW. Type 1 diabetes. Lancet. (2014) 383:69–82. 10.1016/s0140-6736(13)60591-7 23890997 PMC4380133

[2] Kahn SE Cooper ME Del Prato S. Pathophysiology and treatment of type 2 diabetes: perspectives on the past, present, and future. Lancet. (2014) 383:1068–83. 10.1016/s0140-6736(13)62154-6 24315620 PMC4226760

[3] Yuan X Song F Zhang L. Trend analysis of diabetic mortality. Lancet. (2019) 393:1931–2. 10.1016/s0140-6736(18)33051-4 31084957

[4] Zheng Y Ley SH Hu FB. Global aetiology and epidemiology of type 2 diabetes mellitus and its complications. Nat Rev Endocrinol. (2018) 14:88–98. 10.1038/nrendo.2017.151 29219149

[5] Shi GJ Li ZM Zheng J Chen J Han XX Wu J. Diabetes associated with male reproductive system damages: onset of presentation, pathophysiological mechanisms and drug intervention. Biomed Pharmacother. (2017) 90:562–74. 10.1016/j.biopha.2017.03.074 28407577

[6] Gao Y Wei Y Wang Y Gao F Chen Z. *Lycium barbarum*: a traditional Chinese herb and a promising anti-aging agent. Aging Dis. (2017) 8:778–91. 10.14336/ad.2017.0725 29344416 PMC5758351

[7] Masci A Carradori S Casadei MA Paolicelli P Petralito S Ragno R. *Lycium barbarum* polysaccharides: extraction, purification, structural characterisation and evidence about hypoglycaemic and hypolipidaemic effects. A review. Food Chem. (2018) 254:377–89. 10.1016/j.foodchem.2018.01.176 29548467

[8] Tang WM Chan E Kwok CY Lee YK Wu JH Wan CW. A review of the anticancer and immunomodulatory effects of *Lycium barbarum* fruit. Inflammopharmacology. (2012) 20:307–14. 10.1007/s10787-011-0107-3 22189914

[9] Jin M Huang Q Zhao K Shang P. Biological activities and potential health benefit effects of polysaccharides isolated from *Lycium barbarum* L. Int J Biol Macromol. (2013) 54:16–23. 10.1016/j.ijbiomac.2012.11.023 23200976

[10] Xing X Liu F Xiao J So KF. Neuro-protective mechanisms of *Lycium barbarum*. Neuromolecular Med. (2016) 18:253–63. 10.1007/s12017-016-8393-y 27033360

[11] Manthey AL Chiu K So KF. Effects of *Lycium barbarum* on the Visual System. Int Rev Neurobiol. (2017) 135:1–27. 10.1016/bs.irn.2017.02.002 28807155

[12] Wang H Li J Tao W Zhang X Gao X Yong J. *Lycium ruthenicum* studies: molecular biology, phytochemistry and pharmacology. Food Chem. (2018) 240:759–66. 10.1016/j.foodchem.2017.08.026 28946340

[13] Luo Q Cui X Yan J Yang M Liu J Jiang Y. Antagonistic effects of *Lycium barbarum* polysaccharides on the impaired reproductive system of male rats induced by local subchronic exposure to 60Co-gamma irradiation. Phytother Res. (2011) 25:694–701. 10.1002/ptr.3314 21077258

[14] Xin YF You ZQ Gao HY Zhou GL Chen YX Yu J. Protective effect of *Lycium barbarum* polysaccharides against doxorubicin-induced testicular toxicity in rats. Phytother Res. (2012) 26:716–21. 10.1002/ptr.3633 22016089

[15] Qian L Yu S. Protective effect of polysaccharides from *Lycium barbarum* on spermatogenesis of mice with impaired reproduction system induced by cyclophosphamide. Am J Reprod Immunol. (2016) 76:383–5. 10.1111/aji.12558 27572525

[16] Tang ZY Sun D Qian CW Chen Q Duan SS Sun SY. *Lycium barbarum* polysaccharide alleviates nonylphenol exposure induced testicular injury in juvenile zebrafish. Int J Biol Macromol. (2017) 104(Pt. A):618–23. 10.1016/j.ijbiomac.2017.06.035 28636878

[17] Lau BW Lee JC Li Y Fung SM Sang YH Shen J. Polysaccharides from wolfberry prevents corticosterone-induced inhibition of sexual behavior and increases neurogenesis. PLoS ONE. (2012) 7:e33374. 10.1371/journal.pone.0033374 22523540 PMC3327693

[18] Shi GJ Zheng J Han XX Jiang YP Li ZM Wu J. *Lycium barbarum* polysaccharide attenuates diabetic testicular dysfunction via inhibition of the PI3K/Akt pathway-mediated abnormal autophagy in male mice. Cell Tissue Res. (2018) 374:653–66. 10.1007/s00441-018-2891-1 30073544

[19] Shi GJ Zheng J Wu J Qiao HQ Chang Q Niu Y. Beneficial effects of *Lycium barbarum* polysaccharide on spermatogenesis by improving antioxidant activity and inhibiting apoptosis in streptozotocin-induced diabetic male mice. Food Funct. (2017) 8:1215–26. 10.1039/c6fo01575a 28225103

[20] Shi GJ Zheng J Wu J Qiao HQ Chang Q Niu Y. Protective effects of *Lycium barbarum* polysaccharide on male sexual dysfunction and fertility impairments by activating hypothalamic pituitary gonadal axis in streptozotocin-induced type-1 diabetic male mice. Endocr J. (2017) 64:907–22. 10.1507/endocrj.EJ16-0430 28794341

[21] Kitada M Koya D. SIRT1 in type 2 diabetes: mechanisms and therapeutic potential. Diabetes Metab J. (2013) 37:315–25. 10.4093/dmj.2013.37.5.315 24199159 PMC3816131

[22] Mostafa T Nabil N Rashed L Makeen K El-Kasas MA Mohamaed HA. Seminal SIRT1 expression in infertile oligoasthenoteratozoospermic men with varicocoele. Andrology. (2018) 6:301–5. 10.1111/andr.12462 29359516

[23] Mostafa T Nabil N Rashed L Abo-Sief AF Eissa HH. Seminal SIRT1-oxidative stress relationship in infertile oligoasthenoteratozoospermic men with varicocele after its surgical repair. Andrologia. (2020) 52:e13456. 10.1111/and.13456 31696601

[24] Liu C Song Z Wang L Yu H Liu W Shang Y. Sirt1 regulates acrosome biogenesis by modulating autophagic flux during spermiogenesis in mice. Development. (2017) 144:441–51. 10.1242/dev.147074 28003215

[25] El-Mesallamy HO Gawish RA Sallam AM Fahmy HA Nada AS. Ferulic acid protects against radiation-induced testicular damage in male rats: impact on SIRT1 and PARP1. Environ Sci Pollut Res Int. (2018) 25:6218–27. 10.1007/s11356-017-0873-6 29243149

[26] Conde E Alegre L Blanco-Sanchez I Saenz-Morales D Aguado-Fraile E Ponte B. Hypoxia inducible factor 1-alpha (HIF-1 alpha) is induced during reperfusion after renal ischemia and is critical for proximal tubule cell survival. PLoS ONE. (2012) 7:e33258. 10.1371/journal.pone.0033258 22432008 PMC3303832

[27] Dodd MS Sousa Fialho MDL Montes Aparicio CN Kerr M Timm KN Griffin JL. Fatty acids prevent hypoxia-inducible factor-1alpha signaling through decreased succinate in diabetes. JACC Basic Transl Sci. (2018) 3:485–98. 10.1016/j.jacbts.2018.04.005 30175272 PMC6115650

[28] Chen Y Zhang Y Ji H Ji Y Yang J Huang J. Involvement of hypoxia-inducible factor-1alpha in the oxidative stress induced by advanced glycation end products in murine Leydig cells. Toxicol In Vitro. (2016) 32:146–53. 10.1016/j.tiv.2015.12.016 26743761

[29] Joo HY Yun M Jeong J Park ER Shin HJ Woo SR. SIRT1 deacetylates and stabilizes hypoxia-inducible factor-1alpha (HIF-1alpha) via direct interactions during hypoxia. Biochem Biophys Res Commun. (2015) 462:294–300. 10.1016/j.bbrc.2015.04.119 25979359

[30] Xia G Xin N Liu W Yao H Hou Y Qi J. Inhibitory effect of *Lycium barbarum* polysaccharides on cell apoptosis and senescence is potentially mediated by the p53 signaling pathway. Mol Med Rep. (2014) 9:1237–41. 10.3892/mmr.2014.1964 24549741

[31] Luo Q Li J Cui X Yan J Zhao Q Xiang C. The effect of *Lycium barbarum* polysaccharides on the male rats reproductive system and spermatogenic cell apoptosis exposed to low-dose ionizing irradiation. J Ethnopharmacol. (2014) 154:249–58. 10.1016/j.jep.2014.04.013 24746483

[32] Brunet A Sweeney LB Sturgill JF Chua KF Greer PL Lin Y. Stress-dependent regulation of FOXO transcription factors by the SIRT1 deacetylase. Science. (2004) 303:2011–5. 10.1126/science.1094637 14976264

[33] Jiang X Chen J Zhang C Zhang Z Tan Y Feng W. The protective effect of FGF21 on diabetes-induced male germ cell apoptosis is associated with up-regulated testicular AKT and AMPK/Sirt1/PGC-1alpha signaling. Endocrinology. (2015) 156:1156–70. 10.1210/en.2014-1619 25560828 PMC6285187

[34] Coussens M Maresh JG Yanagimachi R Maeda G Allsopp R. Sirt1 deficiency attenuates spermatogenesis and germ cell function. PLoS ONE. (2008) 3:e1571. 10.1371/journal.pone.0001571 18270565 PMC2216432

[35] Jia L Li W Li J Li Y Song H Luan Y. *Lycium barbarum* polysaccharide attenuates high-fat diet-induced hepatic steatosis by up-regulating SIRT1 expression and deacetylase activity. Sci Rep. (2016) 6:36209. 10.1038/srep36209 27824080 PMC5099939

[36] Zhu Y Sun Y Guan W Ya Y Zhang W Bai L. (2016). *Lycium barbarum* polysaccharides enhances SIRT1 expression and decreases MMP-9 and HIF-1alpha expressions in hypoxic pulmonary vascular smooth muscle cells. Xi Bao Yu Fen Zi Mian Yi Xue Za Zhi. 32, 906–910. 27363270

[37] McCreight LJ Bailey CJ Pearson ER. Metformin and the gastrointestinal tract. Diabetologia. (2016) 59:426–35. 10.1007/s00125-015-3844-9 26780750 PMC4742508

[38] Bonnet F Scheen A. Understanding and overcoming metformin gastrointestinal intolerance. Diabetes Obes Metab. (2017) 19:473–81. 10.1111/dom.12854 27987248

